# The genomic and transcriptomic landscapes of clock genes reveal the significance of circadian rhythm in the progression and immune microenvironment of metastatic colorectal cancer

**DOI:** 10.1002/ctm2.755

**Published:** 2022-03-16

**Authors:** Long Zhang, Yikuan Chen, Choon Seng Chong, Xiaoji Ma, Shanyou Tong, Xingfeng He, Yiwei Li, Sanjun Cai, Shaobo Mo, Junjie Peng, Xiang Hu

**Affiliations:** ^1^ Department of Colorectal Surgery Fudan University Shanghai Cancer Center Shanghai China; ^2^ Department of Oncology Shanghai Medical College Fudan University Shanghai China; ^3^ Cancer Institute Fudan University Shanghai Cancer Center Fudan University Shanghai China; ^4^ Department of Colorectal Surgery National University Hospital Singapore

Dear Editor,

Colorectal cancer (CRC) is one of the most common cancers worldwide, of which metastasis is the predominant cause of cancer mortality.^1^ The overall survival (OS) of patients suffering from metastatic CRC (mCRC) has been improved by tailoring systemic therapy to the molecular and pathologic features,^1^ and immunotherapy achieves the longest extension of OS. However, this subset only covers 5% of mCRC patients with high levels of microsatellite instability (MSI‐H) or mismatch repair deficiency (dMMR).^1^, ^2^ Thus, further understanding of the immune microenvironment is critical for expanding the efficacy of immunotherapy in mCRC.^2^

Circadian rhythm fine‐tunes a wide range of biological processes around the clock,^3^ and disruption of circadian rhythm leads to cancer development.^4^ Circadian rhythm has also been shown to modify the immune microenvironment in other cancers,^5^, ^6^ but the role of circadian clock genes in the mCRC immune microenvironment is unclear. Given the associations among circadian rhythm, cancer, and the immune environment, clinical mCRC treatment, especially immunotherapy, could substantially benefit from specific circadian timing of therapy, cancer chronotherapy, which could potentially optimize traditional cancer therapies and even enhance new anticancer drugs.^7^, ^8^ Therefore, circadian clock genes might modify the mCRC immune microenvironment, and understanding this relationship might provide insight for mCRC treatment, especially immunotherapy.

Here, we profiled the genomic landscape of clock genes using multidimensional omics data, including genomics, epigenomics, transcriptomics, pharmacogenomics and clinical survival data, in mCRC derived from The Cancer Genome Atlas (TCGA) (Table 1), Gene Expression Omnibus (GEO) (Table 2) and in‐house experiments. First, the association between clock genes and the metastasis pathways in CRC was analyzed, and it was revealed that multiple oncogenic pathways and clock genes were tightly tethered (Figure 1; Figure S1). For instance, retinoic acid receptor‐related orphan receptor alpha (RORA) and retinoic acid receptor‐related orphan receptor beta (RORB) activate epithelial‐mesenchymal transition (EMT), while RORC inhibits EMT (Figure 1A–C). Notably, some interactions between clock genes and pathways could only be observed in rectal adenocarcinoma (READ) or colon adenocarcinoma (COAD) (Figure 1C; Figure S1C).

**TABLE 1:** Demographics and clinical characteristics of metastatic colorectal cancer patients in TCGA

**Characteristics**	**Level**	**Number (%)**
Age at diagnosis	Mean ± SD	66.4 ± 12.3
	Median (IQR)	68.0 (58.5–76.0)
	<60	127 (27.5)
	≥60	334 (72.5)
Gender	Female	206 (44.6)
	Male	255 (55.4)
Site	Colon	325 (70.4)
	Rectum	136 (29.6)
Histological type	Adenocarcinoma	396 (85.9)
	Mucinous adenocarcinoma	55 (11.9)
	Unknown	10 (2.2)
LNH	<12	48 (10.4)
	≥12	387 (83.9)
	Unknown	26 (5.7)
Lymphatic invasion	Yes	169 (36.6)
	No	252 (54.7)
	Unknown	40 (8.7)
Venous invasion	Yes	97 (21.0)
	No	304 (65.9)
	Unknown	60 (13.1)
Perineural invasion	Yes	47 (10.1)
	No	123 (26.7)
	Unknown	291 (63.2)
T stage	T1	19 (4.1)
	T2	80 (17.4)
	T3	322 (69.8)
	T4	38 (8.3)
	Unknown	2 (0.4)
N stage	N0	265 (57.5)
	N1	115 (25.0)
	N2	80 (17.3)
	Unknown	1 (0.2)
M stage	M0	363 (78.7)
	M1	56 (12.1)
	Unknown	42 (9.2)
TNM stage	Stage I	83 (18.0)
	Stage II	169 (36.7)
	Stage III	140 (30.3)
	Stage IV	57 (12.4)
	Unknown	12 (2.6)
MSI status	MSI‐H	76 (16.5)
	MSS	385 (83.5)

Abbreviations: SD, standard deviation; IQR, interquartile range; LNH, lymph node harvested; MSI, microsatellite instability; MSI‐H, microsatellite instability‐High; MSS, microsatellite stability.

**TABLE 2:** Demographics and clinical characteristics of metastatic colorectal cancer patients in GSE81558

**Characteristics**	**Level**	**Number (%)**
Age at diagnosis	Mean ± SD	67.3 ± 8.5
	Median (IQR)	67.5 (61.8–75.0)
	<60	7 (16.7)
	≥60	35 (83.3)
Gender	Female	12 (28.6)
	Male	30 (71.4)
Site	Liver	19 (45.2)
	Right colon	2 (4.8)
	Left colon	9 (21.4)
	Rectum	12 (28.6)
T stage	T2	1 (2.4)
	T3	34 (80.9)
	T4	7 (16.7)
N stage	N0	13 (31.0)
	N1	21 (50.0)
	N2	8 (19.0)
Metastatic status	Synchronous	30 (71.4)
	Metachronous	12 (28.6)

Abbreviations: SD, standard deviation; IQR, interquartile range.

**FIGURE 1:**
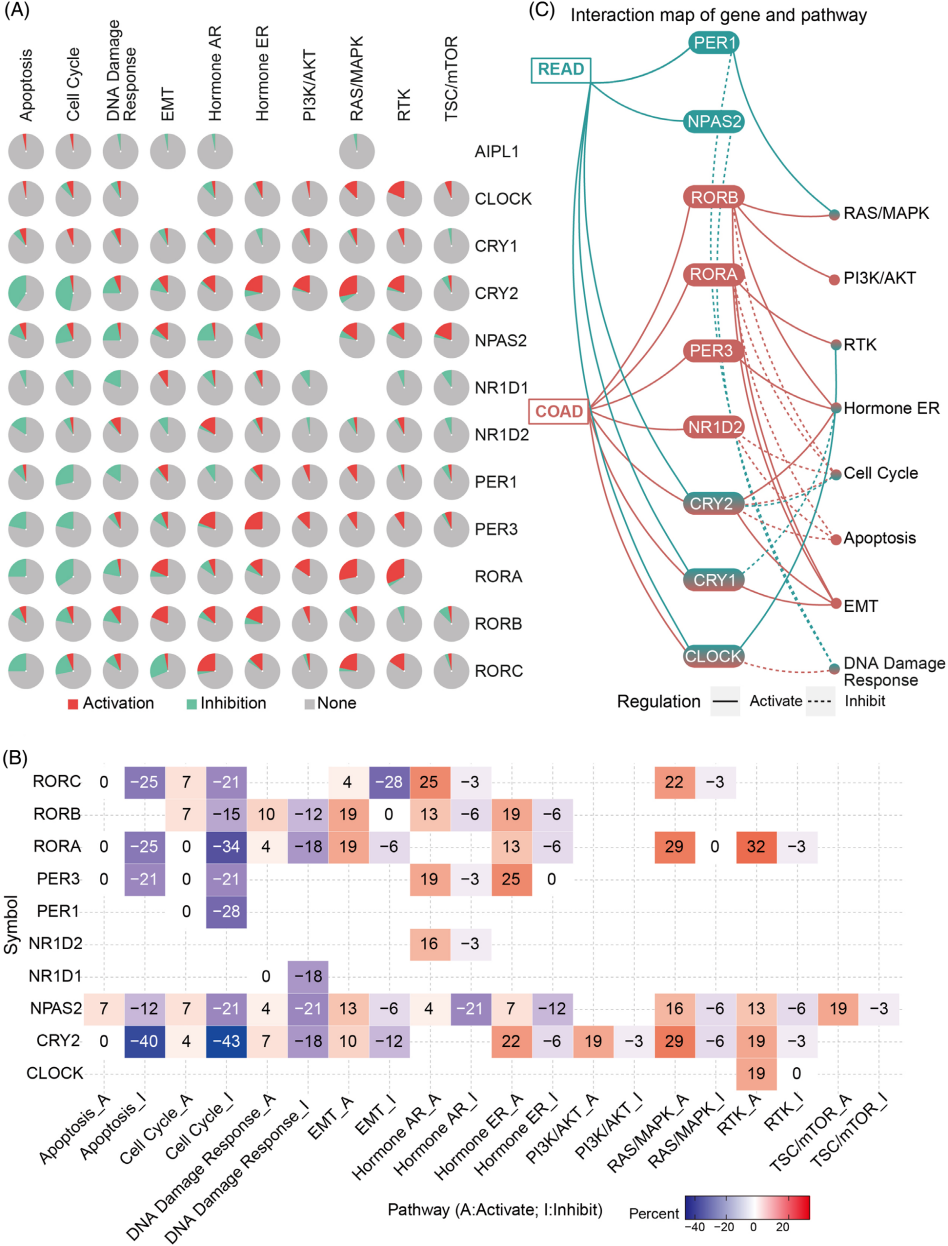
Functional effects of core clock genes in metastatic pathways. (A) Global percentage of signalling in which a clock gene has effect on this pathway. (B) Heatmap showing clock genes that have function (inhibit or activate). (C) This network shows the relationship between clock genes and pathways by a line connection. The solid line means activation, and the dashed line means inhibition. The number of patients with colorectal cancer (*n* = 461)

As alterations of clock genes might result in CRC metastasis, we used the SW480 COAD cell line and its metastatic counterpart SW620 to gain further evidence. Compared with SW480 cells, SW620 cells displayed a delayed, even reverse circadian rhythm oscillations. The oscillations of core clock genes were robustly diminished (Figure 2A1–8; Figure S2), and the correlations of clock genes were markedly changed (Figure 2B). Accordingly, analysis of the clock genes in metastasis free (mFree CRC) and mCRC tissues showed a similar pattern to that of the two cell lines, and even more changes could be observed (Figure 2C).

**FIGURE 2:**
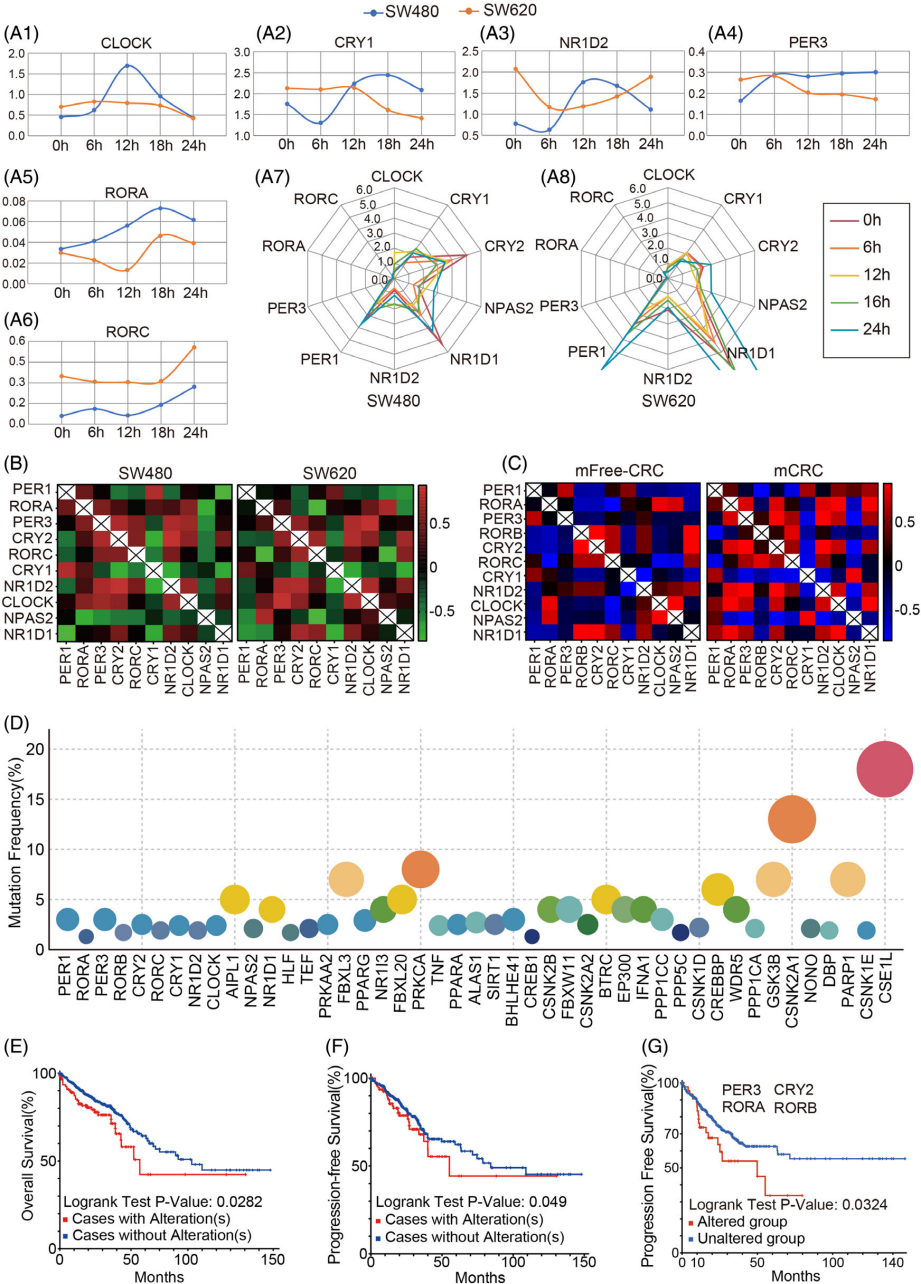
Disturbance and alterations of clock genes in metastatic colorectal cancer . (A1–A6) Time course for transcriptional expression of core clock genes in primary SW480 cells (blue) and metastatic SW620 cells (orange). (A7–A8) The circadian rhythm oscillations of clock genes in SW480 and SW620. (B) Heat maps of spearman correlation between each pair of core clock genes for SW480 and SW620 cells, *n* = 2. (C) Heat maps of spearman correlation between each pair of core clock genes for metastasis free CRC (mFree CRC) and metastatic CRC (mCRC) (*n* = 447). (D) The number of mutations (point size) and frequency of mutations (*Y*‐axis) for each clock gene. Kaplan–Meier curves showing (E) overall survival and (F) progression‐free survival between patients with (red) or without (blue) mutations in core clock genes and (G) critical mutated genes

We further explored whether alterations of clock genes (including genetic mutations and expression changes) that modulate CRC metastasis could affect the prognosis of CRC patients. Different clock genes display distinct mutation frequencies in CRC, and patients bearing mutations in core clock genes had significantly worse OS and progression‐free survival. Notably, patients bearing even low‐frequency mutations of the core circadian genes (RORA, period circadian protein homolog 3 (PER3), RORB and cryptochrome‐2 (CRY2)) showed significantly poor PFS (Figure 2D–G; Figure S3).

Profiling the distribution of methylation and gene expression across the clock genes revealed that methylation status and transcriptome expression showed an overall negative correlation (Figure S4; Figure S5A,B). Therefore, we analyzed the transcriptional patterns of clock genes in mCRC using GEO data containing paired RNA sequencing and found that the clock genes were transcriptionally altered when CRC metastasized (Figure 3A). Not only was there an association between clock gene expression and the stage of CRC (Figure S5C1–6), but there was also a potential association between clock gene expression and the metastasis status of CRC (Figure S5D1–8). Further determination of the prognostic significance of clock genes in CRC revealed that high expression of CLOCK predicted significantly better PFS and OS, while high expression of NR1D1 and PER3 predicted significantly worse PFS and OS (Figure 3B–G). NPAS2, RORB and other clock genes also showed prognostic significance (Figure S6).

**FIGURE 3:**
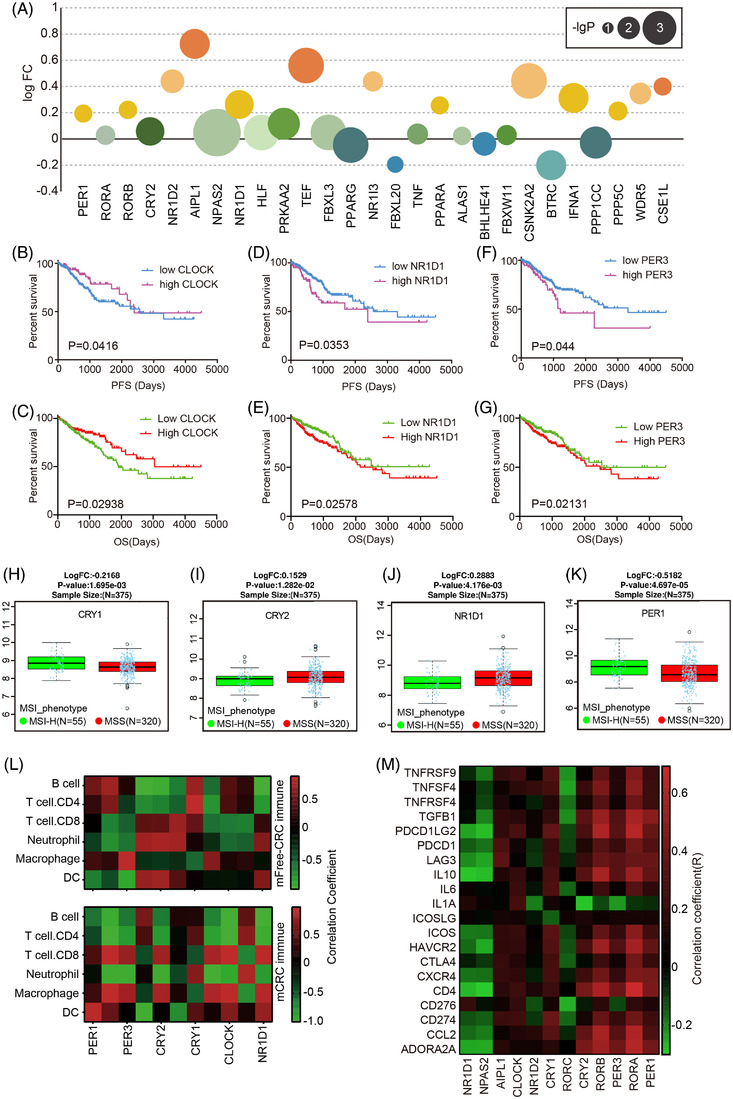
The transcriptional dysregulation and immune correlation of clock genes in metastatic colorectal cancer. (A) fold change for each clock gene compared with paired primary samples. The number of patients with liver metastasis (*n* = 19), and primary colorectal cancer (*n* = 23). (B–G) Association between clock genes’ expression and progression‐free survivals (PFS) and overall survivals (OS). (H–K) Individual cases for clock genes associated with the microsatellite instability (MSI) status of colorectal cancer (CRC) patients, MSI‐H: MSI‐high; MSS: microsatellite stable. (L) Heat maps of correlation between each core clock gene and immune cells. (M) Heat maps of spearman correlation between each pair of core clock genes and immune‐related genes

For the application of immunotherapy to mCRC patients, MSI status is clinically decisive, and clock genes were reported to be associated with the immune microenvironment. Therefore, we examined whether clock genes could be associated with CRC MSI status. Surprisingly, CRY1, CRY2, NR1D1 and PER1 were significantly associated with CRC MSI status (Figure 3H–K). The investigation of the correlations between clock genes and immune infiltration showed that mCRC showed a distinct pattern compared with mFree CRC (Figure 3L), suggesting that the change in clock genes would change infiltrating immune cells in CRC to modulate CRC metastasis. Analysis of the expression of clock genes and immune factors also showed that clock genes had significant correlations with immune related genes (Figure 3M). To more directly assess the effects of circadian rhythm on the CRC immune status, we analyzed the role of clock genes in immune cell infiltration in tumour tissues. Significant correlations between core clock genes and immune cells were observed. For instance, CLOCK is positively correlated with CD4+ and CD8+ TILs but negatively correlated with macrophages and neutrophils. These findings are consistent with the survival prognosis analysis (Figure 3B,C).

Tumour subtype classification, including molecular classification (Epstein‐Barr virus (EBV), microsatellite instability (MSI), hypermutated‐single‐nucleotide variant (HM‐SNV), chromosomal instability (CIN), and genome stable (GS)) and immune subtypes (C1: wound healing, C2: IFNγ dominant, C3: inflammatory, C4: lymphocyte depleted, C5: immunologically quiet and C6: TGF‐β dominant), often provides essential insights into stratifying patients for immunotherapy response.^9^, ^10^ We found that the expressions of CLOCK, CRY1 and NPAS2 were significantly changed in the C6 subtype (Figure 4B,D,H) but not in the molecular classification subtypes. The findings suggest the potential roles of clock genes as markers for specific immune subtypes of CRC (Figure 4). Finally, investigation of The Cancer Therapeutics Response Portal and Genomics of Drug Sensitivity in Cancer projects showed that clock genes could be drug targets (Figures S7 and S8), which suggests the potential of combining cancer chronotherapy and immunotherapy for better outcomes of mCRC patients. In conclusion, this study, through genomic landscape analysis, reveals the significance of circadian clock genes in mCRC metastasis, and prognosis, especially immune microenvironment modulation, and provides preliminary evidence for the potential of using cancer chronotherapy for the regimens of mCRC immunotherapy.

**FIGURE 4:**
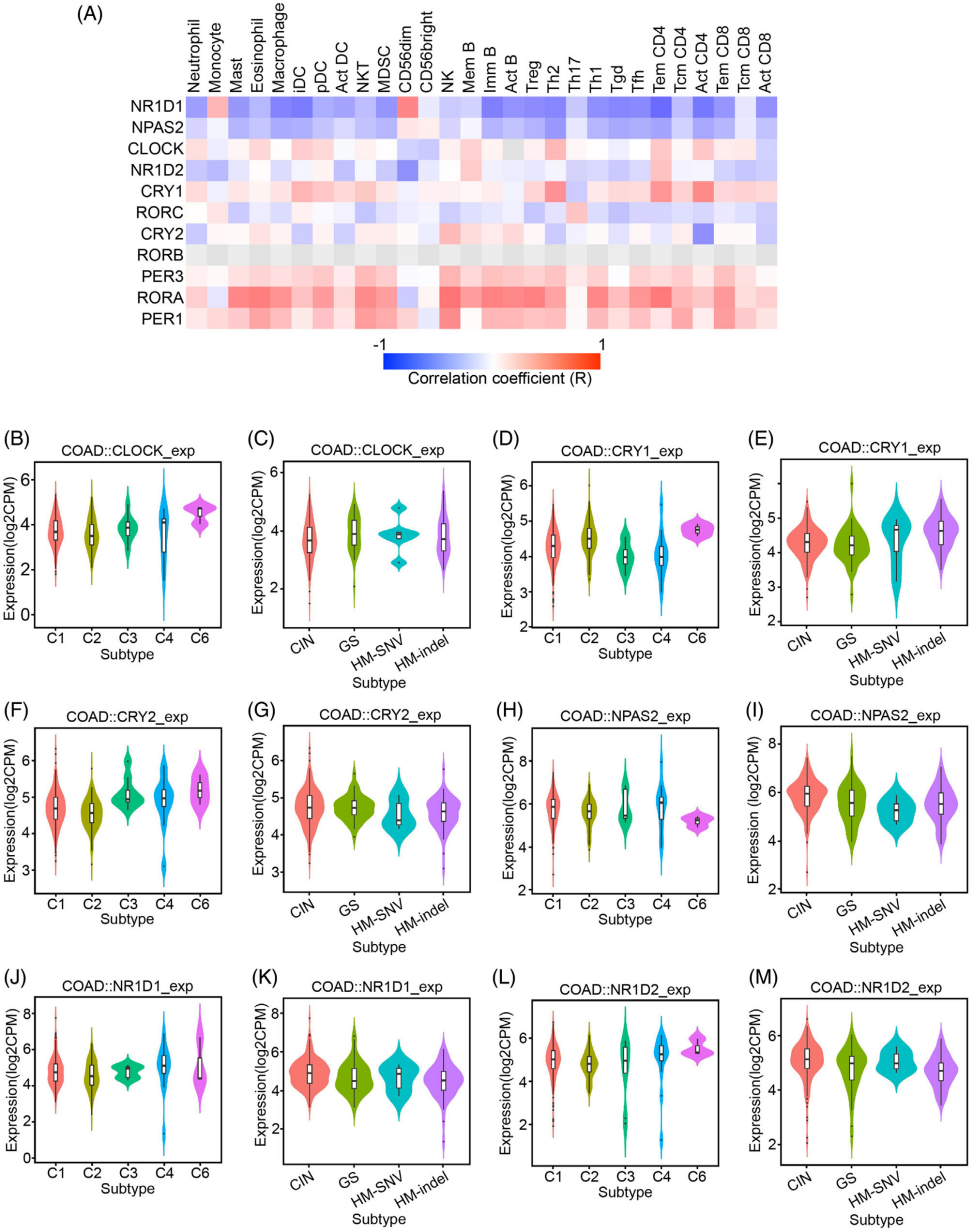
Interactions between immune microenvironment and clock genes. (A) Heat maps of relations between abundance of tumour‐infiltrating lymphocytes (TILs) and clock genes expression. (B–M) Clinically relevant clock genes across different molecular classification and immune subtypes (*n* = 461)

## CONFLICT OF INTEREST

The authors declare no conflict of interest.

## Supporting information

Supporting Information

Supporting Information
