# Supplementary material for: The genomic and transcriptomic landscapes of clock genes reveal the significance of circadian rhythm in the progression and immune microenvironment of metastatic colorectal cancer
Source: Clin Transl Med. 2022 Mar 16;12(3):e755. doi: 10.1002/ctm2.755 (PMC8926903; doi:10.1002/ctm2.755)
Supplement: Supplementary file 1 — Supporting Information [file CTM2-12-e755-s002.pdf]

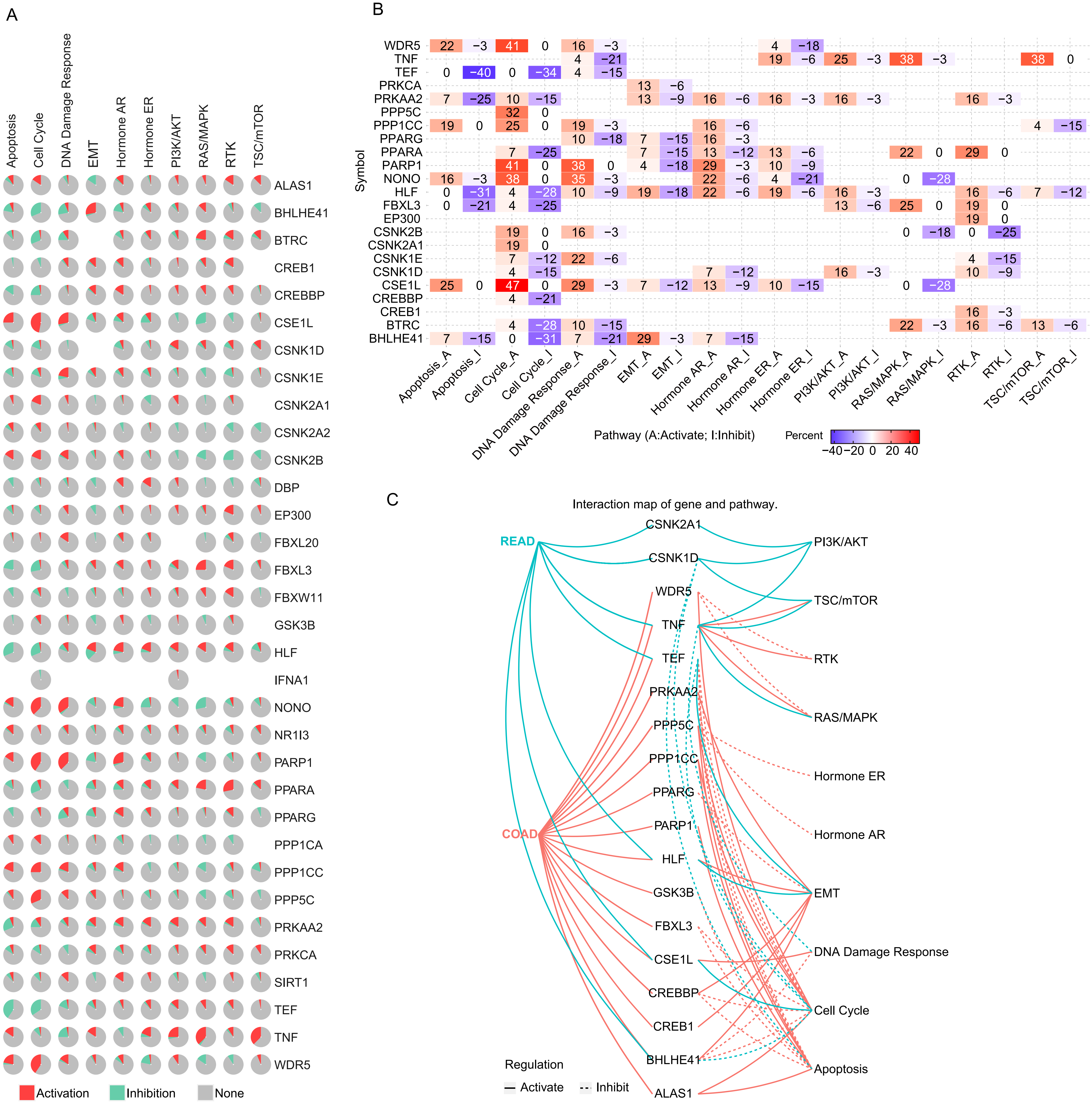

Supplementary Figure S1. Related to Figure 1. Functional effects of clock-associated genes in metastatic pathways. (A) Global percentage of signaling in which a gene has effect on this pathway. (B) Heat map showing genes that have function (inhibit or activate). (C) This network showing the relationship between genes and pathways by a line connection. Solid line means activation, dashed lines means inhibition.

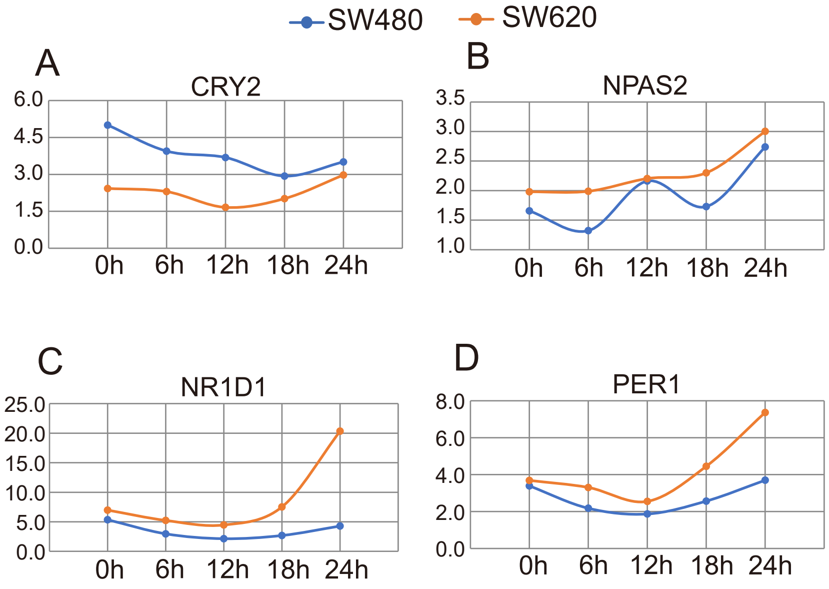

Supplementary Figure S2. Related to Figure 2A1-A6. Time course for transcriptional expression of core clock genes in primary SW480 cells (blue) and metastatic SW620 cells (orange).

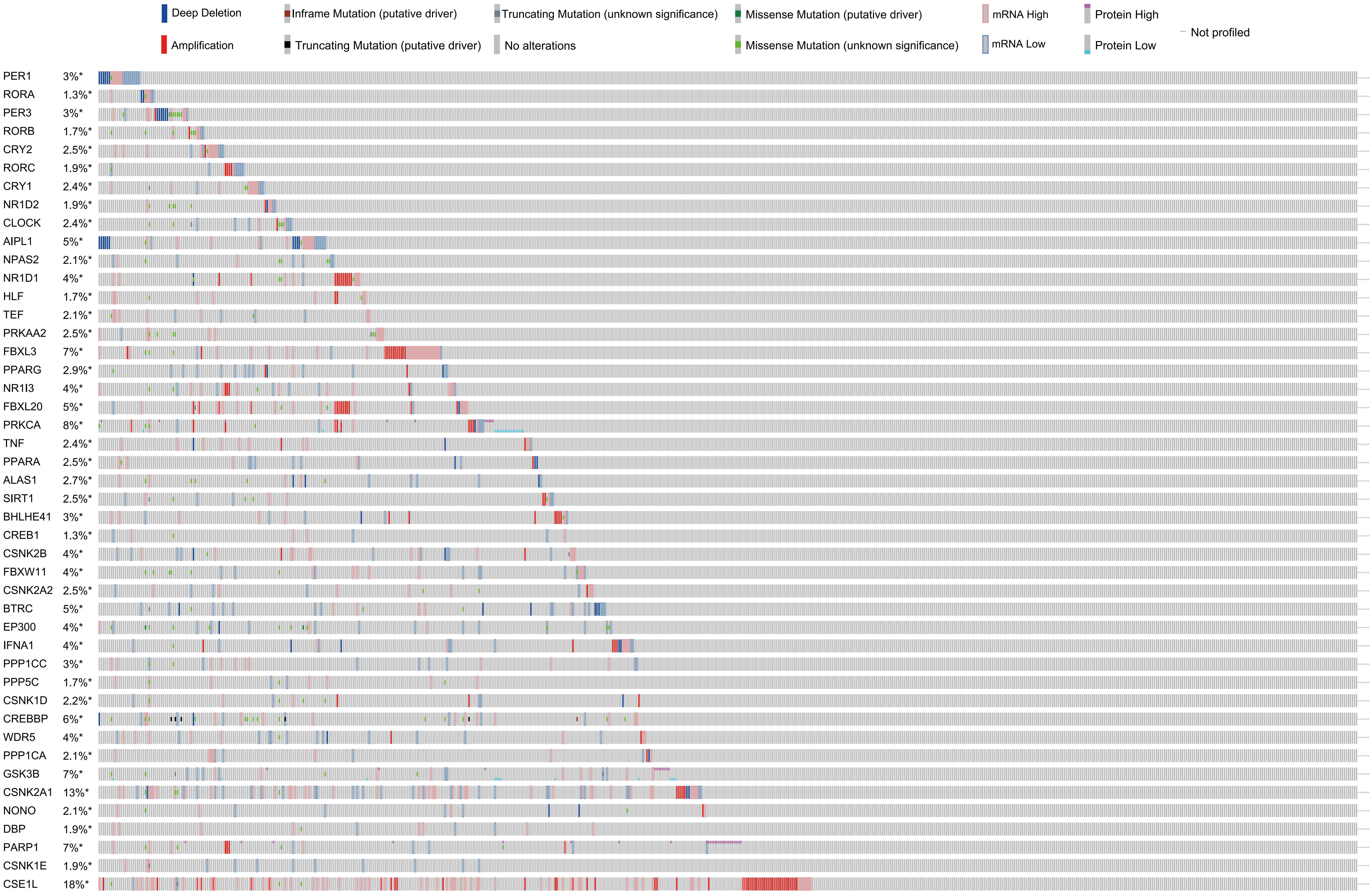

Supplementary Figure S3. Related to Figure 2D. Mutational landscape of clock genes in metastatic CRC.

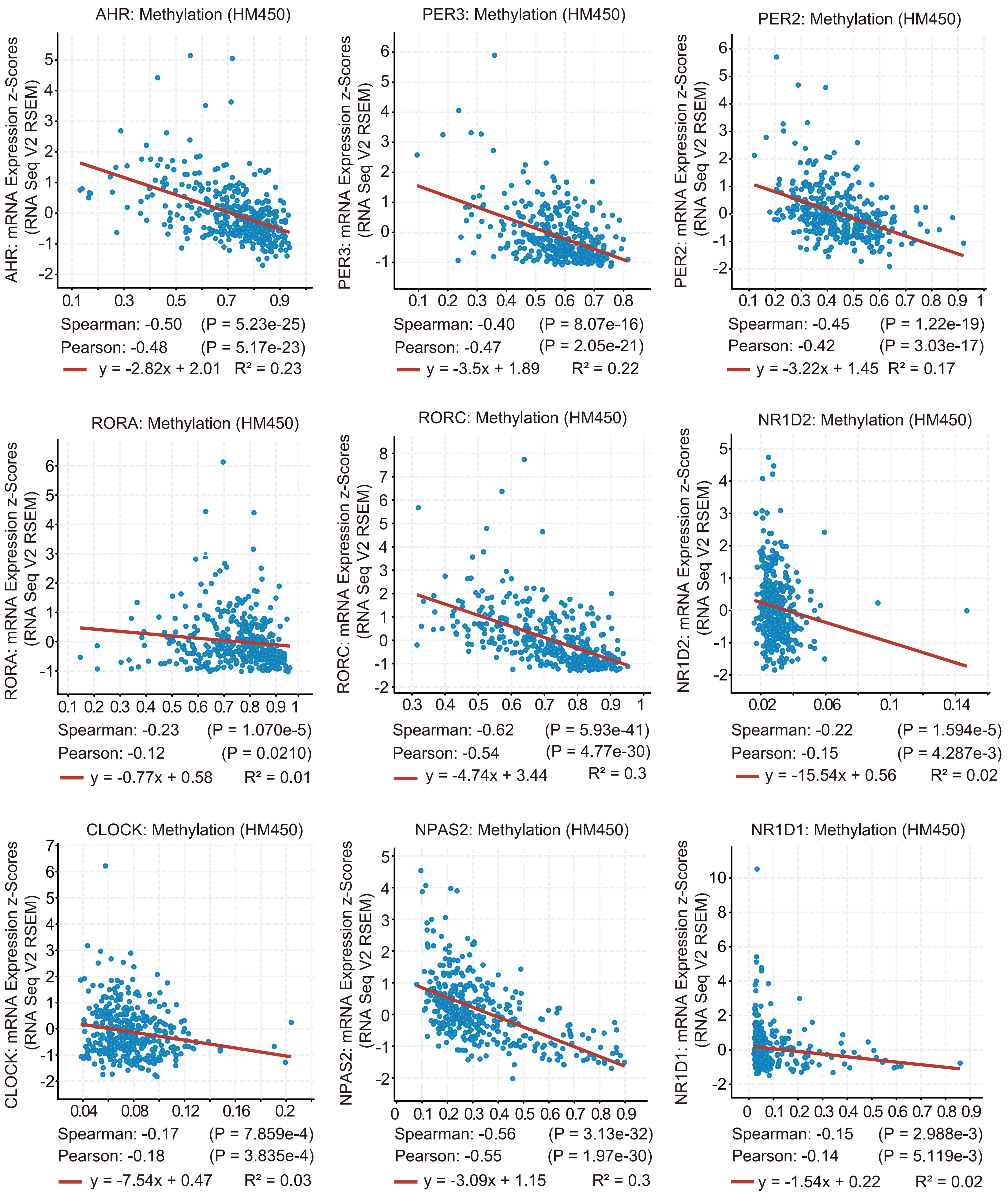

Supplementary Figure S4. Negative correlation between promoter DNA methylation levels and gene expression.

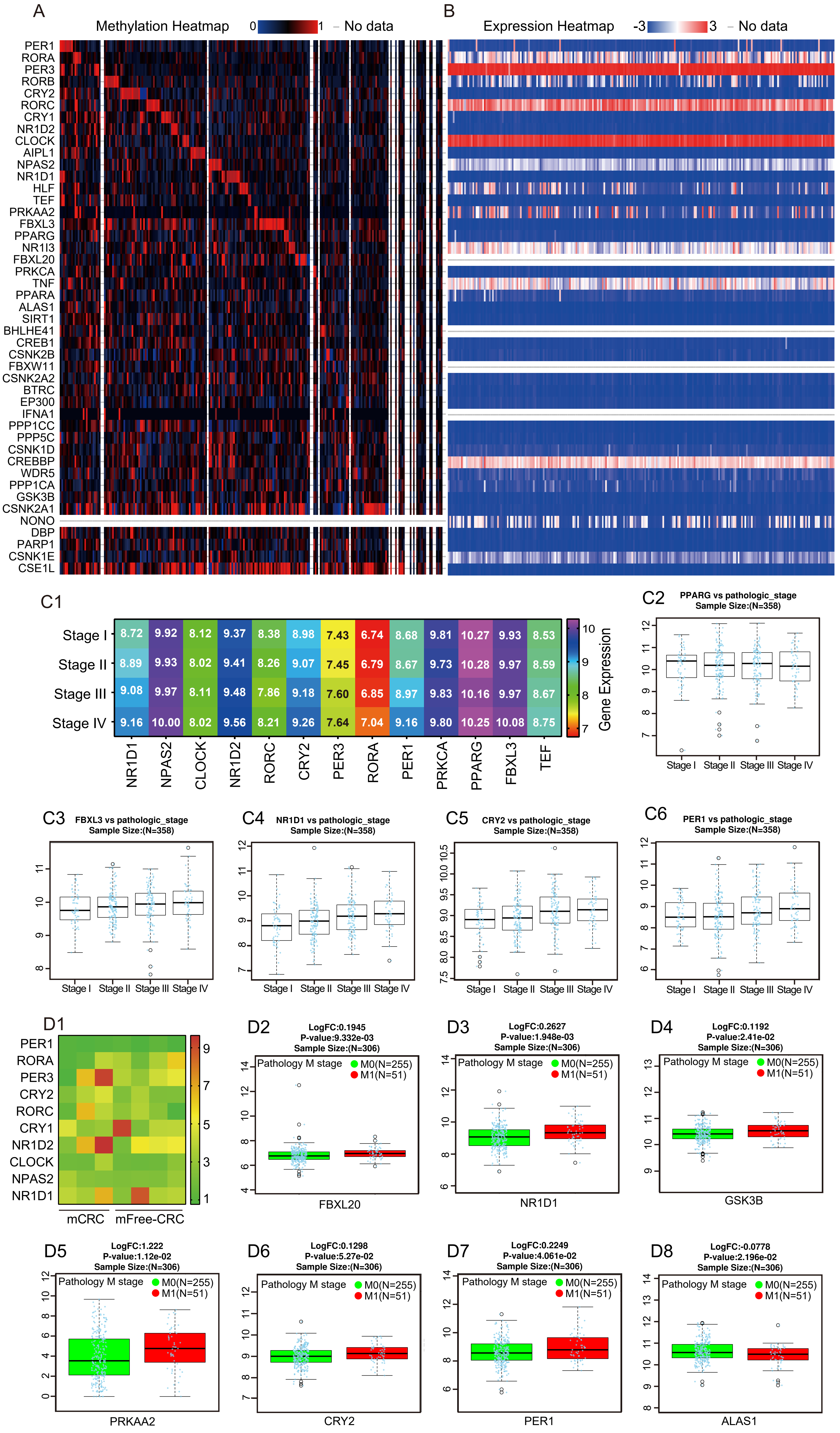

Supplementary Figure S5. (A) Heat map showing correlation between promoter DNA methylation levels and (B) gene expression across clock genes (n=461). (C1) Heat map showing clock genes' levels expressed differentially among tumor stages (n=461). (C2-C6) Individual cases for clock genes associated with CRC stages. (D1) Transcriptional expression of core clock genes in mFree-CRC (n=4) and mCRC (n=3). (D2-D8) Individual cases for clock genes associated with CRC pathology M stage.

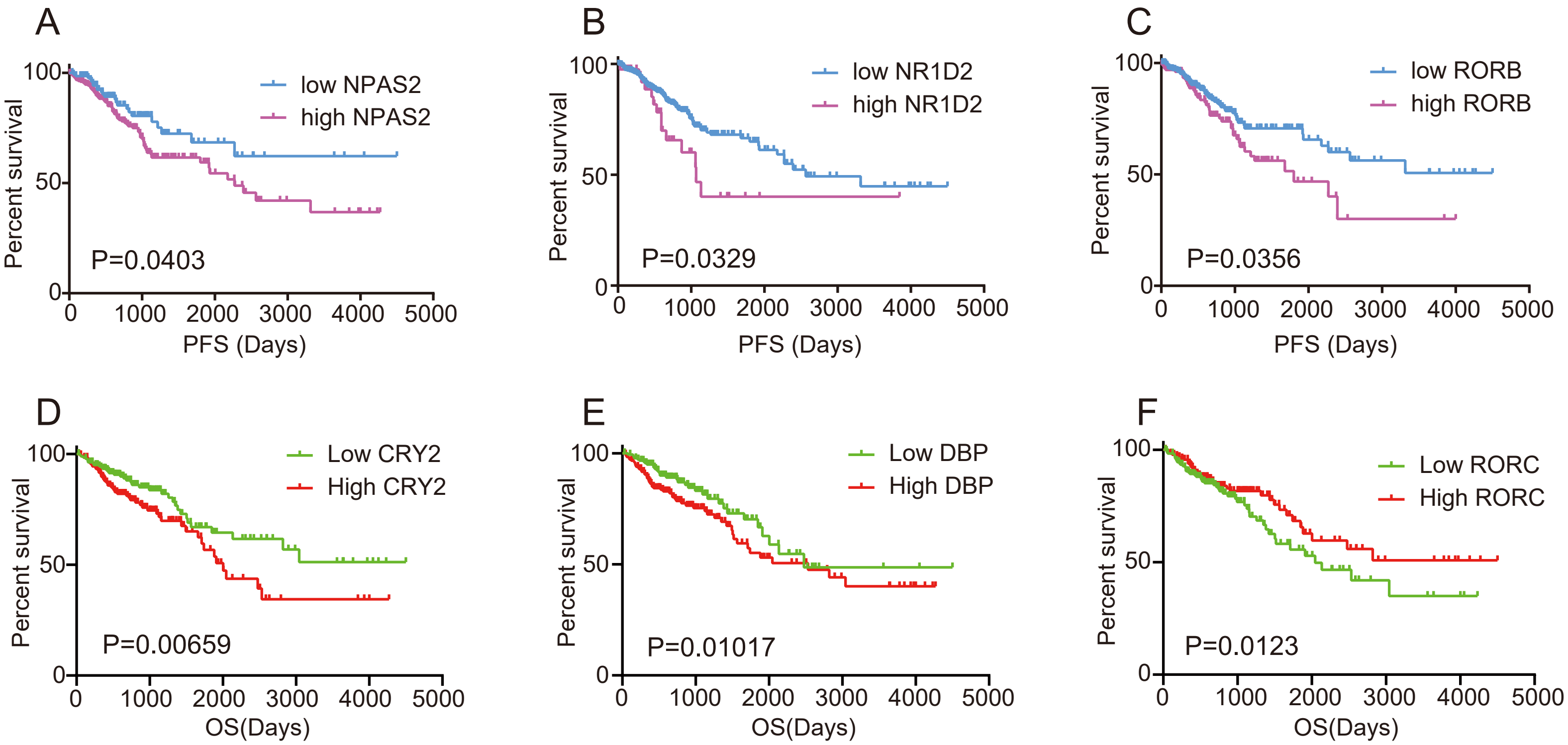

Supplementary Figure S6. Related to Figure 3B-G. (A-F) Association between clock genes' expression and progression-free survivals (PFS) or overall survivals (OS).

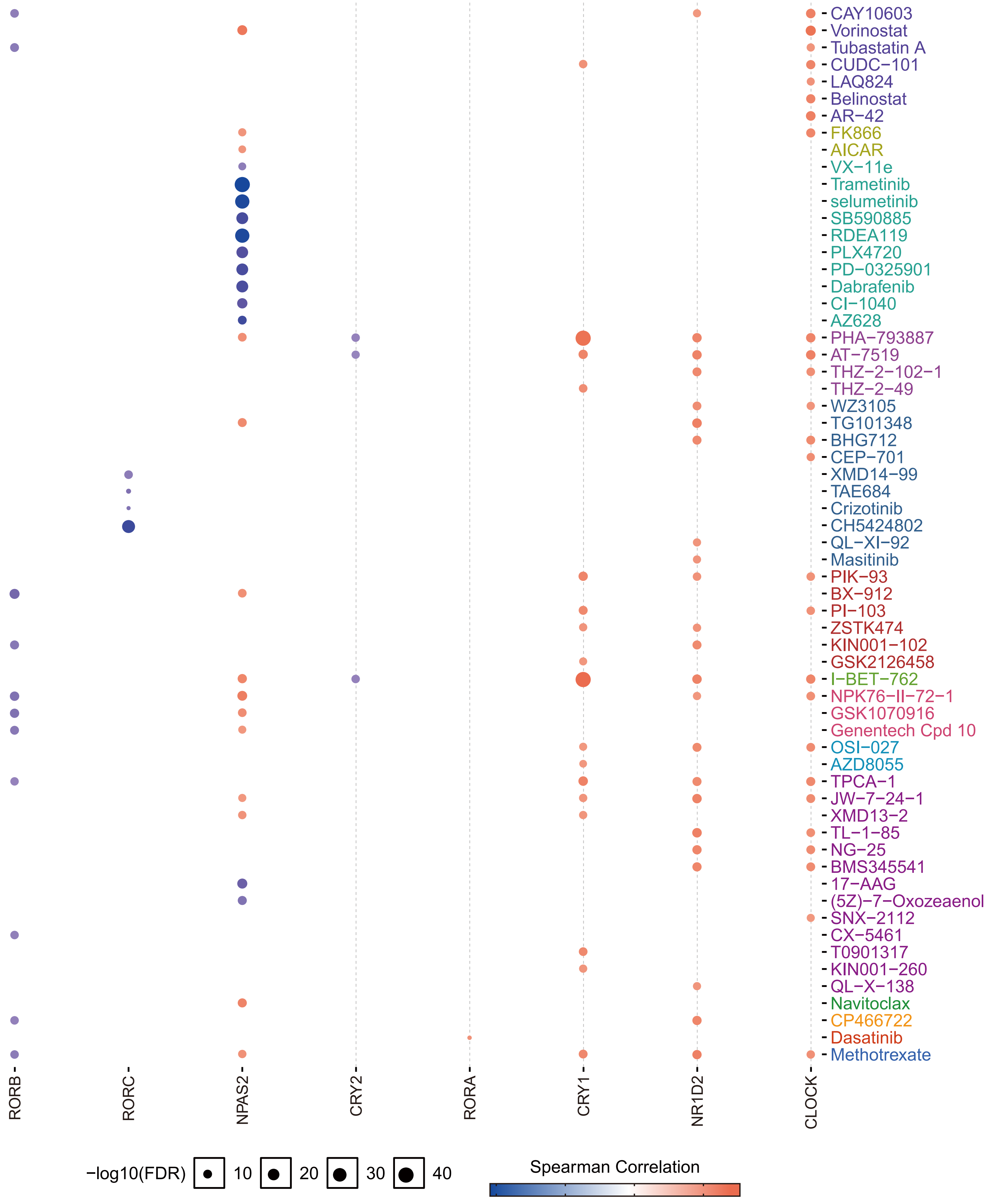

Supplementary Figure S7. Potential therapeutics targeted to clock genes. Correlation between drug sensitivity (area under curves, AUCs) and gene expression of core clock genes.

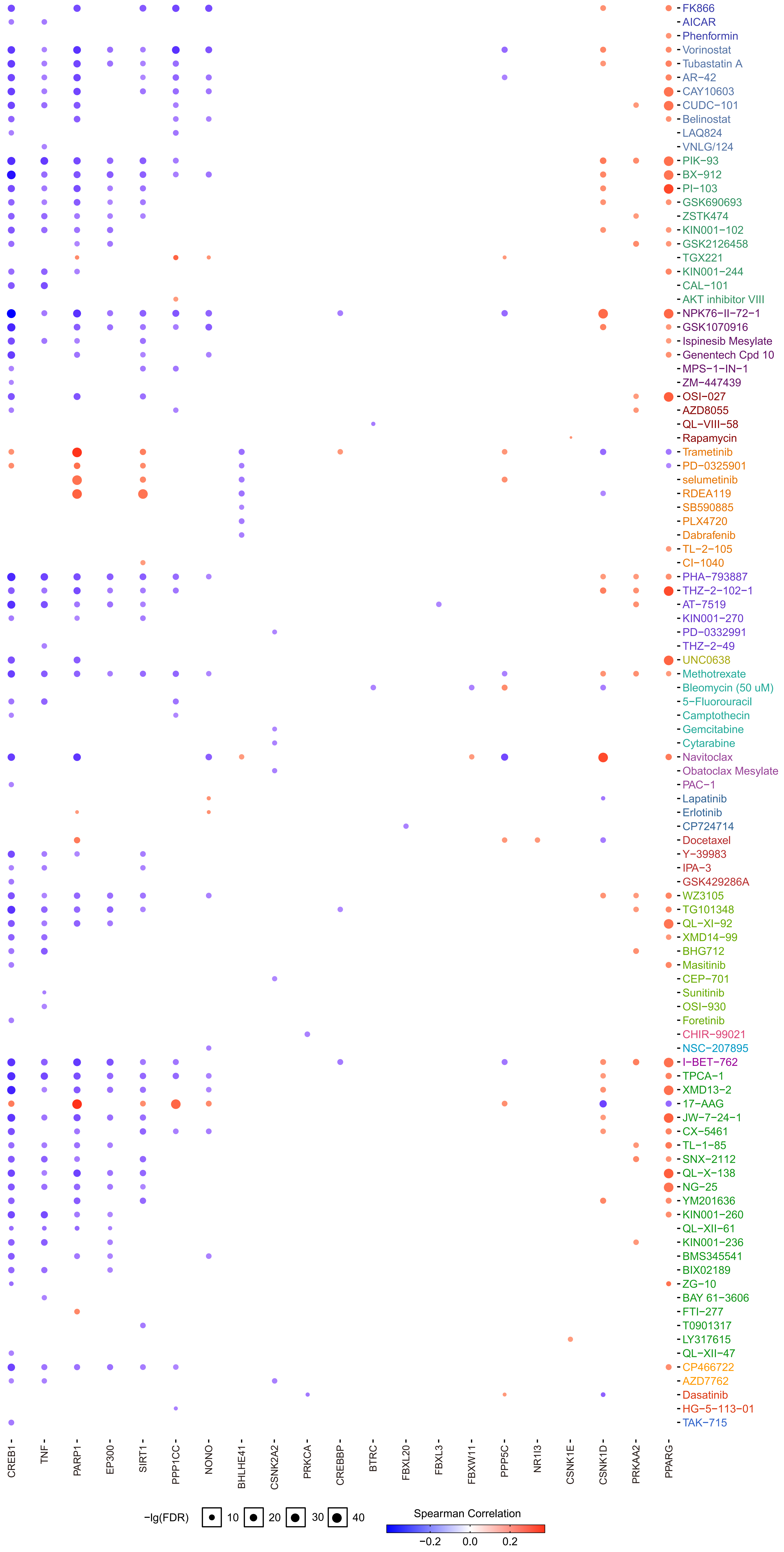

Supplementary Figure S8. Related to Supplementary Figure S7. Potential therapeutics targeted to clock genes. Correlation between drug sensitivity (area under curves, AUCs) and gene expression of core clock genes.
