# Supplementary material for: The genomic and transcriptomic landscapes of clock genes reveal the significance of circadian rhythm in the progression and immune microenvironment of metastatic colorectal cancer
Source: Clin Transl Med. 2022 Mar 16;12(3):e755. doi: 10.1002/ctm2.755 (PMC8926903; doi:10.1002/ctm2.755)
Supplement: Supplementary file 2 — Supporting Information [file CTM2-12-e755-s001.docx]

**Supporting information for**

**The genomic and transcriptomic landscapes of clock genes reveal the significance of circadian rhythm in the progression and immune microenvironment of metastatic colorectal cancer**

Long Zhang*^1,2,4^ Yikuan Chen*^1,2^, Choon Seng CHONG*^3^, Xiaoji Ma^1,2^, Shanyou Tong, Xingfeng He, Yiwei Li^1,2^, Sanjun Cai^1,2,4^, Shaobo Mo^1,2^, Junjie Peng^1,2^, Xiang Hu^1,2^

1. Department of Colorectal Surgery, Fudan University Shanghai Cancer Center, Shanghai, 20032, China

2. Department of Oncology, Shanghai Medical College, Fudan University, Shanghai 200032, China

3. Department of Colorectal Surgery, National University Hospital, Singapore 119288

4. Cancer institute, Fudan University Shanghai Cancer Center, Fudan University, Shanghai 200032, China

**Methods:**

**Patient specimens and cell lines**

CRC tissues were obtained from patients diagnosed and treated in Fudan University Shanghai Cancer Center (FUSCC). There were four patients without relapse for at least 5 years follow-up, while another three patients suffered from metastasis. Written informed consent were obtained at the enrollment. The research protocol was reviewed and approved by the ethics committee of the FUSCC. SW480 colon cancer cell line and its metastatic counterpart SW620^1^ were obtained from Type Culture Collection Cell Bank, Chinese Academy of Sciences and were cultured with Dulbecco's modified Eagle's medium (DMEM) supplemented with 10% fetal bovine serum (FBS) at 37℃ and 5% CO_2_.

**RNA extraction**

For in-house experiments, RNA samples were obtained from SW480 cells, SW620 cells and CRC tissues respectively using TRIzol reagent (Thermo Fisher Scientific) as the manufacture instructed. For cells, they were harvested at a confluence of 70-80%. After discarding the medium, cells were washed by PBS for twice. Then PBS was discarded and TRIzol reagent was added to the culture plate for cell lysis. For CRC tissues, the samples were washed twice by PBS and cut into small pieces. Then the samples were resuspended by TRIzol reagent and grinded in a grind miller at a temperature under 0 ℃. The suspensions obtained by lysing cells from cell culture plates and CRC tissue samples using TRIzol reagent were treated as follows. Chloroform was added into the TRIzol suspension at a ratio of 1:5 (v:v). Then the mixture was thoroughly mixed and centrifuged at 12,000 g for 10 min at 4℃. The supernatant was preserved and equal volume of isopropanol was added following thorough mix and incubation. Then the mixture was centrifuged at 12,000 g for 10 min at 4℃ for RNA precipitation. The RNA precipitation was washed by 70% ethanol diluted by DEPC-treated water for twice. After volatilization of the 70% ethanol, the RNA precipitation was resolved by DEPC-treated water. Then the concentration and the quality of the RNA were checked, followed by RNA sequencing.

**Sample preparation for time-course RNA Sequencing**

SW480 and SW620 cells were plated in doubles in 6-well plates for synchronizing by medium change. Next day, RNA extraction was performed for samples obtained every 6 h in 24 hours. The Illumina HiSeq 2000 was performed for High-throughput sequencing with biological replicates^2^. Fragments per kilobase per million reads (FPKM) were used for the related analyses in Figure 2 and Supplementary Figure S2.

**mRNA, protein expression and signaling pathways in TCGA and GEO**

Normalized gene expression information for relapse free CRC data were obtained from The Cancer Genome Atlas (TCGA) database available from UCSC Xena, Cancer Genomics Browser of the University of California Santa Cruz (<https://genome-cancer.ucsc.edu/>) and analyzed as previously reported^3^. Genes were considered for differentially expressed between primary tumor and paired liver metastasis samples in microarray expression data from Gene Expression Omnibus (GEO) (GSE81558) were normalized using Robust Multichip Average^4^ and were considered ^5^.

The Cancer Proteome Atlas^6^ (<http://tcpaportal.org/tcpa/>download.html) was used to evaluated protein expression with reverse phase protein array (RPPA). The ten key signaling pathways involvement were assessed by pathway score based on protein expression, which was calculated as the sum of the relative protein levels of all positive regulatory components minus the equivalent sum for the negative regulatory components ^7^. Interactive effects of clock genes were evaluated by Student *t* tests for statistical difference significance in pathway score with P value < 0.05. With statistical significance, the level of individual clock genes positively correlated with the pathway score suggests activation of the pathway, in contrast, the negative correlation suggests inhibition of the pathway.

**Disturbance of clock genes in mCRC**

To analyze the association between clock genes and metastatic pathways, we used GSCALite (http://bioinfo.life.hust.edu.cn/web/GSCALite/)^8^ for the analysis. To evaluate the disturbance of clock genes in mCRC, we counted the number of pathways correlated with clock genes in CRC metastatic lesions minus that in primary tumor samples. Correlation between the transcriptional level of clock genes and other key pathways were calculated on the basis of Pearson’s analysis, and considered significant if FDR < 0.05.

**Mutation and DNA methylation analysis**

The mutation data and DNA methylation status data were obtained from the TCGA data portal (https://www.cbioportal.org/).The number and frequency of non-silent somatic mutations in every clock gene were calculated in CRC. To assess the effect of promoter DNA methylation on clock gene mRNA expression, the methylation probes comprised of from-1,500 bp to +500 bp around transcription start sites (TSS), and generated pair wise heat maps of methylation and gene mRNA expression. Spearman’s correlation between promoter DNA methylation and clock gene expression was performed and retained the minimum Spearman correlation coefficient.

**The clinical relevance of circadian rhythm**

Clinical information for CRC with relapse data, overall survival, disease stages, and tumor subtypes were obtained from TCGA database available from UCSC Xena, Cancer Genomics Browser of the University of California Santa Cruz (https://genome-cancer.ucsc.edu/). A log-rank test was performed to predict the role of clock genes in overall and relapse-free survivals. Transcriptional expression levels were cut off by the median level or the optimal cutoff values calculated by X-tile^9^. *ANOVA* was performed to detect clock genes with differential levels among different tumor classification and TNM stages.

**Correlation of drug sensitivity and clock genes**

Genomics of Drug Sensitivity in Cancer (GDSC) (<http://www.cancerrxgene.org/downloads>) project was designed to identify the drug response for 1,000 cancer cell lines. Normalized transcriptional gene expression levels and area under the curves (AUCs) are also included in the dataset. Cancer Therapeutics Response Portal (CTRP) (<https://ocg.cancer.gov/programs/ctd2/data-portal>) have included drug response (AUCs) for 481 small molecules across cancer cell lines. We evaluated the small-molecule sensitivity based on baseline gene expression levels as previously described^10^. Briefly, the Pearson correlation coefficients of genes’ levels and drug response AUC were transformed to normalization using Fisher’s Z. The cutoff values determined as IzI > 3.89 and IzI > 4.13 for drug sensitivity from GDSC and CTRP, respectively.

**Identification of immune relevance for clock genes**

We assessed the correlation between abundance of tumor infiltrating lymphocytes (TILs) and expression levels of clock genes with the help of the TISIDB database ( http://cis.hku.hk/TISIDB)^11^. In all, 28 TILs were calculated based on the immune-related signatures from Charoentong's study^12^. For each TIL, the relative abundance was inferred by gene set variation analysis (GSVA) on the basis of gene expression profile.

**Statistical analysis**

The Wilcoxon signed rank test was utilized to evaluate the statistical significance of the up- or downregulation of genes. Correlation analyses between gene expression and clinical pathological factors were performed using the Spearman correlation. All analyses were corrected for multiple testing by the Bonferroni method. All statistical tests were two-sided and performed using SPSS 22 (Statistical Package for Social Sciences, Chicago, IL). P < 0.05 was considered as significant.

1. Lelbovitz A, Wright WC, Pathak S, Siciliano MJ, Daniels WP. Detection and analysis of a glucose 6-phosphate dehydrogenase phenotype B cell line contamination. *J Natl Cancer Inst*. Sep 1979;63(3):635-45. doi:10.1093/jnci/63.3.635

2. Spies D, Ciaudo C. Dynamics in Transcriptomics: Advancements in RNA-seq Time Course and Downstream Analysis. *Comput Struct Biotechnol J*. 2015;13:469-77. doi:10.1016/j.csbj.2015.08.004

3. Robinson MD, McCarthy DJ, Smyth GK. edgeR: a Bioconductor package for differential expression analysis of digital gene expression data. *Bioinformatics*. Jan 1 2010;26(1):139-40. doi:10.1093/bioinformatics/btp616

4. Irizarry RA, Hobbs B, Collin F, et al. Exploration, normalization, and summaries of high density oligonucleotide array probe level data. *Biostatistics*. Apr 2003;4(2):249-64. doi:10.1093/biostatistics/4.2.249

5. Sayagues JM, Corchete LA, Gutierrez ML, et al. Genomic characterization of liver metastases from colorectal cancer patients. *Oncotarget*. Nov 8 2016;7(45):72908-72922. doi:10.18632/oncotarget.12140

6. Li J, Lu Y, Akbani R, et al. TCPA: a resource for cancer functional proteomics data. *Nat Methods*. Nov 2013;10(11):1046-7. doi:10.1038/nmeth.2650

7. Akbani R, Ng PK, Werner HM, et al. A pan-cancer proteomic perspective on The Cancer Genome Atlas. *Nat Commun*. May 29 2014;5:3887. doi:10.1038/ncomms4887

8. Liu CJ, Hu FF, Xia MX, Han L, Zhang Q, Guo AY. GSCALite: a web server for gene set cancer analysis. *Bioinformatics*. Nov 1 2018;34(21):3771-3772. doi:10.1093/bioinformatics/bty411

9. Camp RL, Dolled-Filhart M, Rimm DL. X-tile: a new bio-informatics tool for biomarker assessment and outcome-based cut-point optimization. *Clin Cancer Res*. Nov 1 2004;10(21):7252-9. doi:10.1158/1078-0432.CCR-04-0713

10. Rees MG, Seashore-Ludlow B, Cheah JH, et al. Correlating chemical sensitivity and basal gene expression reveals mechanism of action. *Nat Chem Biol*. Feb 2016;12(2):109-16. doi:10.1038/nchembio.1986

11. Ru B, Wong CN, Tong Y, et al. TISIDB: an integrated repository portal for tumor-immune system interactions. *Bioinformatics*. Oct 15 2019;35(20):4200-4202. doi:10.1093/bioinformatics/btz210

12. Charoentong P, Finotello F, Angelova M, et al. Pan-cancer Immunogenomic Analyses Reveal Genotype-Immunophenotype Relationships and Predictors of Response to Checkpoint Blockade. *Cell Rep*. Jan 3 2017;18(1):248-262. doi:10.1016/j.celrep.2016.12.019
